# Confronting gender stereotypes in sports vocational education: a case study

**DOI:** 10.3389/fspor.2025.1673199

**Published:** 2025-09-16

**Authors:** Celia Marcen, Violeta Gil-Pablo, María Isabel Cuadrado-Santañes, Marta Rapún-López

**Affiliations:** ^1^ Faculty of Human Sciences and Education, Universidad de Zaragoza, Huesca, Spain; ^2^ Faculty of Health Sciences and Sport, Universidad de Zaragoza, Huesca, Spain

**Keywords:** inequality, training, leadership, feminism, spain

## Abstract

This study explores the impact of a training workshop on gender equality among vocational training students in teaching and social and sports activities in Aragon, Spain. The evaluation of attitudes, beliefs and gender stereotypes related to leadership in sports was conducted on the basis of an analysis of pre- and post-intervention surveys. The methodology incorporated participatory techniques such as brainstorming, killer data and gamified interactive tools, with the objective of encouraging critical reflection and active learning. The results indicate that, although no statistically significant changes were observed, there was an improvement in the Egalitarianism Index (+0.51) after the workshop, especially among female participants. Furthermore, cluster analysis revealed a shift towards attitudes that are more homogeneous and favourable towards equality. The study concludes that this type of intervention has the potential to raise awareness among future sports professionals about structural inequalities and gender stereotypes, and highlights the importance of incorporating specific equality content into vocational training curricula. Notwithstanding the constraints imposed by the sample size and duration, this experience is presented as a promising pedagogical proposal that can be replicated in other educational contexts.

## 1 Introduction

The principle of equality between women and men has been a fundamental tenet of the European Union since its establishment. Notwithstanding this fact, women in leadership positions in the majority of the most productive sectors remain a minority and face a wide range of barriers. This phenomenon is evident across various domains, including education, business, and sports, where women are disproportionately represented in lower-ranking positions (1–3).

The Berlin Declaration (4) made it clear that equal opportunities for men and women to benefit from sport are based on gender equality, which is necessarily linked to the equal representation of women and men at all levels of the institutions and entities that organise and regulate sport, and especially in those where decision-making takes place. In this regard, the United Nations considers that equal participation is not only a requirement for improving sports institutions, but also a necessary condition for improving the situation of women in society as a whole.

In the domain of physical and sports activities, there has been a gradual increase in the participation of girls and women in the Spanish sports system. However, a gender gap persists across all age groups. The 2022 survey on sporting habits demonstrates that the gender gap remains significant, with a 11-percentage point discrepancy between male and female respondents. This disparity is further pronounced in the context of federated sports, where the gap extends to 13.5 points (5). These imbalances are also evident in the workplace, with only 11.1% of the 9,000 coaches who attained the highest level of accreditation (level 3) in 2021 being female, accounting for 24.7% of all coaches who received this accreditation.

As posited by Claringbould & Knoppers (6) and further elaborated in the work of Sauleda Martínez et al. por H/M (7), women encounter a multitude of obstacles that impede their ascent to leadership roles. These obstacles, which are more or less invisible and act as a disincentive for those from certain backgrounds to assume leadership positions, have been termed the “glass ceiling” (8). This phenomenon is more prevalent in traditionally male-dominated fields such as sport (9). As indicated by numerous technical reports and scientific studies, the sports labour market has seen an increase in the presence of women, yet they are not adequately represented in sports management or in the sector's decision-making mechanisms (2, 10–13).

In a 2014 study, Burton conducted a systematic review of the reasons why, despite increased opportunities for girls and women in sport, they remain underrepresented in leadership positions at all levels and in all areas of sport (participation, governing bodies, employment in sports companies, federated sport, etc.). The present author identified causes at the macro level, including institutionalised gender practices.

The present study aims to assess the effects on the opinions, beliefs and attitudes regarding gender equality among a group of vocational sports training students who receive specific training on gender equality in sports leadership.

## 2 Pedagogical framework

The sports education system in the Autonomous Community of Aragon (Spain) consists of (14):

• University degrees, with a Bachelor's Degree in Primary Education with a specialisation in Physical Education and a Bachelor's Degree in Physical Activity and Sports Sciences.
• Vocational Education Training (VET) courses, including the basic training cycle in “access and maintenance of sports facilities”, the intermediate training cycle in “nature and leisure guide”, the advanced training cycle in “physical conditioning”, and the advanced training cycle in "socio-sports teaching and animation".
• Special sports education leading to qualifications as Sports Technicians and Advanced Sports Technicians in various disciplines.

The project was carried out with students from the VET programme in "Socio-Sports Teaching and Animation", which is part of the specific higher-level vocational training and lasts 2,000 h. Upon completion, students are awarded the title of Higher Technician in the corresponding field. The studies consist of modules that are taken at the educational centre and another part is training in a workplace (220 h).

ORDER ECD/1352/2018, of 31 July, establishing the curriculum for the Advanced Technical Certificate in Socio-Sports Teaching and Animation for the Autonomous Community of Aragon, establishes in Article 4 that “The general competence of this certificate consists of developing, managing and evaluating recreational physical and sports animation projects for all types of users, programming and directing teaching, socio-sportive inclusion and leisure activities, coordinating the actions of the professionals involved, ensuring safety, respecting the environment and achieving user satisfaction, within the established cost limits” (15): 27846).

And Article 5 of the same specifies the professional, personal and social skills specific to the qualification:

“c) Manage the implementation of the social sports activities project, organising the resources and actions necessary for its promotion, development and supervision.

k) Direct and facilitate the teaching of physical and sports activities, adapting the programme to the dynamics of the activity and the group and evaluating the participants' learning.

l) Direct and facilitate the planned social and sports inclusion activities, adapting them to the dynamics of the activity and the group.

m) Direct and promote recreational physical and sporting activities and scheduled games, adapting them to the dynamics of the activity and the group.

q) Organise and coordinate work teams responsibly, supervising their development, maintaining fluid relationships and assuming leadership, as well as providing solutions to any group conflicts that may arise.

u) Carry out basic management tasks for the creation and operation of a small business and show initiative in their professional activity with a sense of social responsibility” (15): 27846).

In other words, it gives them coordination, management, direction and leadership functions, which are further reinforced by the career opportunities available to them, including sports activities coordinator, physical and sports activities coordinator in sports facilities of tourist companies or public and private entities, coordinator of extracurricular activities in schools, manager of educational leisure projects for children and young people, director of educational leisure activities for children and young people, director of extracurricular activities in schools or head of the tourist entertainment department.

Given the existing gap between men and women in the sports sector, particularly in sports management and administration, where women are in the minority and face a series of structural barriers (16–18), this project proposes specific training on equality in leadership positions in sport, so that students in the VET in Sports become aware of the issue and can be agents of change once they enter the labour market.

The central activity of this project was the design, implementation and evaluation of a training workshop on gender stereotypes in leadership positions in the sports VET students. The design of this project was *ad hoc*, with the objective being to raise awareness of gender issues in sports management. In a participatory manner, seeking reflection and interaction between students and teachers, as well as among students themselves, gamified activities were included using the Mentimenter® and Quizyzz© applications. The incorporation of interactive tools has been demonstrated to enhance student participation, thereby fostering group reflection and active learning (19). These platforms facilitate the collection of responses in real time, thereby enhancing group engagement. Consequently, these tools not only enable participation and interactive learning, but also increase student retention and motivation (20). Gamified activities facilitate learning by promoting intrinsic motivation, active participation, and immediate feedback, which are key elements for building meaningful knowledge. According to Deterding et al. (21), gamification involves the use of game elements in non-playful contexts to enhance student engagement and motivation. This strategy encourages active learning by integrating mechanics such as rewards, challenges and levels, which, according to Kapp (22), stimulates persistence and self-regulation in students. Furthermore, recent research indicates that gamification not only increases motivation but also improves content retention and academic performance (23), making it an effective pedagogical tool for diverse educational environments. In this context, where some of the topics could be considered “hard”, it is even more important not only from the point of view of learning but also of creating a relaxed and playful atmosphere for debate.

The initial technique employed was brainstorming, a widely recognized methodology for problem-solving that emphasizes creativity. This approach facilitated a collaborative evaluation, production and organization of ideas (24). Moreover, the utilization of this participatory instrument at the commencement of the workshop to delineate fundamental concepts facilitated the generation of preliminary knowledge among the participants. This information can be used as an initial assessment, allowing for the adaptation of teaching in real time, with adjustments to the language and dynamics to suit the needs of the students (25).

Another highly effective technique employed was “killer data”, a powerful technique in data communication with a strong social component, especially useful for highlighting inequalities that often go unnoticed. The term “killer data” is used to denote data that, rather than being “hard data” due to its statistical significance, is characterized by its ability to provoke a strong emotional or cognitive reaction (26). The data indicates that:

1. This phenomenon is indicative of a structural injustice or hidden imbalance.
2. This phenomenon is not intuitive or easily perceived without evidence.
3. The objective is to elicit a sense of astonishment or a paradigm shift in the audience's perspective.

The efficacy of this approach is rooted in its ability to challenge the prevailing attitudes of “this doesn't affect me” or “I don't see it in my environment” (27).

The structure of the workshop was: First, there was a brainstorming session on what the students knew about the “gender gap”, followed by the concepts of “gender equality” and “gender equity”, or “gender bias”. Based on this discussion, real statistics on the incorporation of women into different areas of sport were presented, and the levels of management and leadership at the regional, national and international levels were explored in depth. The final part of the workshop was devoted to the concept of leadership in sport, female leadership and what is required to improve the participation of women in decision-making positions in sport.

In order to assess gender stereotypes pre and post training workshop among students in vocational sports training, a questionnaire was designed containing a section on sociodemographic data, participation in sports, gender stereotypes in everyday tasks, gender stereotypes in leadership positions, and ideological self-placement. The following questionnaires, which have been demonstrated to possess a high degree of reliability, were utilized as a point of reference for the design of this questionnaire (adaptations were made to the questions when necessary):

• Survey on the Perception of Equality between Men and Women and Gender Stereotypes (28).
• SPORTnet4Women for the Women Leaders in Sport programme (29).

Due to the size of the sample and the treatment of this study as a case study, a questionnaire was validated by expert judgement, a procedure used to ensure the content validity of the instrument, i.e., that its items adequately and pertinently represent the construct to be measured. This process consists of submitting the questionnaire to a panel of specialists in the field, who analyse aspects such as the clarity, relevance, consistency and adequacy of the questions (30). In this case, three experts in three areas (sports management, physical activity and sports sciences, and sociology) carried out this validation, and the proposed modifications were made.

The variables included sociodemographic data (gender, age, educational level, and employment status) and main variables related to attitudes towards equality and shared responsibility (items on caregiving tasks, perception of equality, and approval of institutional measures such as paternity leave). In the first phase, descriptive analyses (frequencies, means, and standard deviations) were performed to characterise the sample. Subsequently, pre- and post-scores were compared using Student's t-tests for related samples, and differences between groups (men/women) were examined using Student's t-tests for independent samples. An overall equality index was also calculated for the whole sample and for subgroups, and variations between pre- and post- were explored. To identify attitude profiles, cluster analyses were performed, comparing the composition of the groups before and after the intervention. All analyses were performed with computational support in Python (version 3.10), using standard libraries for data processing and statistical testing (pandas, scipy, scikit-learn, among others).

Following the receipt of favourable approval from the Research Ethics Committee of the Autonomous Community of Aragon (CEICA) with reference C.P.- C.I. PI24/088, the pre-questionnaire was distributed 10 days before the workshop, when the students had no information about the project or its theme. The post-questionnaire was completed after the training.

## 3 Learning environment

The project was carried out at the Integrated Public Vocational Training Centre (CPIFP) Pirámide in Huesca (Spain), a publicly owned centre financed with public funds. This educational centre is located on the outskirts of Huesca, 3.5 km from the city centre. It receives students from the city (60%) and from different villages in the Hoya de Huesca and Monegros region (40%), thus mixing rural and urban populations. Its training activities include initial vocational training (500 students in 14 intermediate and advanced vocational training courses), job placement and reintegration activities for workers, and continuing education for the working population. It incorporates a career guidance and information service and another service for assessing skills acquired through other non-formal learning and work experience. The Centre has large outdoor areas, sports facilities and a residence for students from outside the area.

This experience was conducted with the students of the 1st year of the Socio-Sports Teaching and Animation VET Advanced programme. Twenty-two students participated in this study, of whom 16 were male and 6 were female, all of them aged 18–25. Six of them were working at the time of the study (they were student-workers), three had experience in coordination in sports organisations (two in the federation sector and one in a sports company), and three in sports management (one in formal sports training and two in the sports federation sector). Most consider themselves to be in good health (with an average score of 4 out of 5) and in good shape (3.8 out of 5 on average). And 50% would like to pursue a career in sports management in the future, 31.8% are not sure, and the remaining 18.2% are not interested in doing so.

## 4 Results

### 4.1 VET students’ opinions, attitudes and beliefs regarding gender equality in leadership positions

After calculating the descriptive statistics for the sample, a Pearson correlation analysis was performed to measure the strength and direction of the linear relationship between the different sociodemographic variables and the questionnaire items, finding the following correlations:

a. Gender shows positive correlations with several items that reflect gender stereotypes:
  1. “Women cannot be aggressive..”: *r* = 0.51
  2. “Women are less capable of learning mathematical skills..”: *r* = 0.50
  3. “Men will have more leadership skills..”: *r* = 0.54
b. Employment status negatively correlated with items reflecting gender biases, for example: “Women are less capable…”: *r* = −0.42
c. Self-assessed health and physical fitness have moderate positive correlations with some leadership and stereotype items. For example, better health correlates with believing that “pregnancy should be considered for management positions” (*r* = 0.49).
d. Sports coordination has positive correlation with: “Women have the ability to acquire leadership skills” (*r* = 0.46).

To quantify participants' orientation toward gender equality, we created an Egalitarianism Index. This index was computed by averaging the scores of four Likert-scale items that directly reflect egalitarian beliefs: “Women have the capacity to acquire the necessary skills to be successful leaders”, “Men and women should have the same opportunities to participate in executive training programs”, “The lack of women in leadership positions is a social and structural issue”, and “Women have the objectivity required to make sound business decisions”. Each item was rated on a 1–7 scale (from strong disagreement to strong agreement), and the resulting index reflects the mean value of these items. Higher scores on the index represent stronger egalitarian attitudes.

To determine which personal and contextual factors are associated with egalitarian beliefs, we performed a multiple linear regression using the constructed Egalitarianism Index as the dependent variable. The model included gender, employment status, self-rated health and physical fitness, involvement in sports management, and political orientation.

The analysis revealed that being male, being employed, and rating oneself as physically fit were significantly associated with lower egalitarian scores. Interestingly, gender had the strongest negative effect (*β* = −2.42, *p* = .044), suggesting that male participants tended to express less support for gender equality in leadership roles. In contrast, better self-rated health was marginally associated with more egalitarian attitudes (*β* =  +1.40, *p* = .054). Other factors, such as involvement in sports management or political ideology, did not show statistically significant effects in this model, though the direction of coefficients suggests avenues for future exploration.

To explore underlying patterns in participants' responses, it was conducted a Principal Component Analysis (PCA) using the items of the questionnaire. PCA is a statistical technique used to reduce the dimensionality of complex datasets while retaining the most relevant information. It does so by transforming the original variables into a smaller set of new, uncorrelated variables called principal components. In our case, the first two principal components accounted for a substantial proportion of the total variance (PC1 = 31.5%, PC2 = 18.2%). These components allowed us to visualize and interpret participant positioning within a two-dimensional space, facilitating the detection of meaningful clusters.

The interpretation of each principal component was based on the loadings of the original items (Table 1). For instance, items related to gender equality beliefs loaded strongly on PC1, suggesting this component represents a dimension of egalitarian attitudes. Meanwhile, items linked to career aspirations and leadership potential contributed notably to PC2, indicating a motivational or ambition-related dimension.

**Table 1:** Principal component analysis (PCA).

Item	PC1 loading	PC2 loading
In general, I would say that your current state of health is…	0.231	0.324
In general, I would say that your current physical condition is…	0.195	0.336
I am willing to work hard to progress in sports management positions	0.331	−0.218
My professional goal is to promote sports management positions	0.328	−0.249
I have given serious consideration to pursuing a career in sports management at the highest level	0.311	−0.205
Balancing high-level management work with family life is a very difficult task	0.017	−0.424
It is less desirable for women than for men to have a job that requires responsibility	0.066	−0.202
Women have the objectivity required to properly evaluate business situations	−0.268	0.037
Challenges at work are more important to men than to women	−0.346	0.109
Women have the ability to acquire the skills necessary to be successful managers	−0.138	0.079
Men and women should have equal opportunities to participate in management training programmes	−0.111	0.057
The possibility of pregnancy should be taken into account when deciding on a senior management position	0.279	0.266
Women cannot be aggressive in business situations that require it	−0.087	0.312
Women are less capable of learning mathematical and mechanical skills than men	0.239	0.296
In general, men will have more of the skills required for a leadership position, as this has always been the case	0.203	0.341
On average, female managers are less capable than men of contributing to an organisation's overall objectives	−0.127	−0.012
The fact that there are not as many women in jobs is a social and structural problem.	−0.259	0.124
Self-placement on the ideological scale (1 being furthest to the left and 10 being furthest to the right).	0.316	0.017

This analysis revealed that participants could be meaningfully grouped according to their latent response patterns—capturing complex interrelations among variables that might not emerge from individual item analyses alone. The PCA thus served as the foundation for the subsequent cluster analysis, enhancing the interpretability and theoretical coherence of the typologies identified.

**Figure 1:**
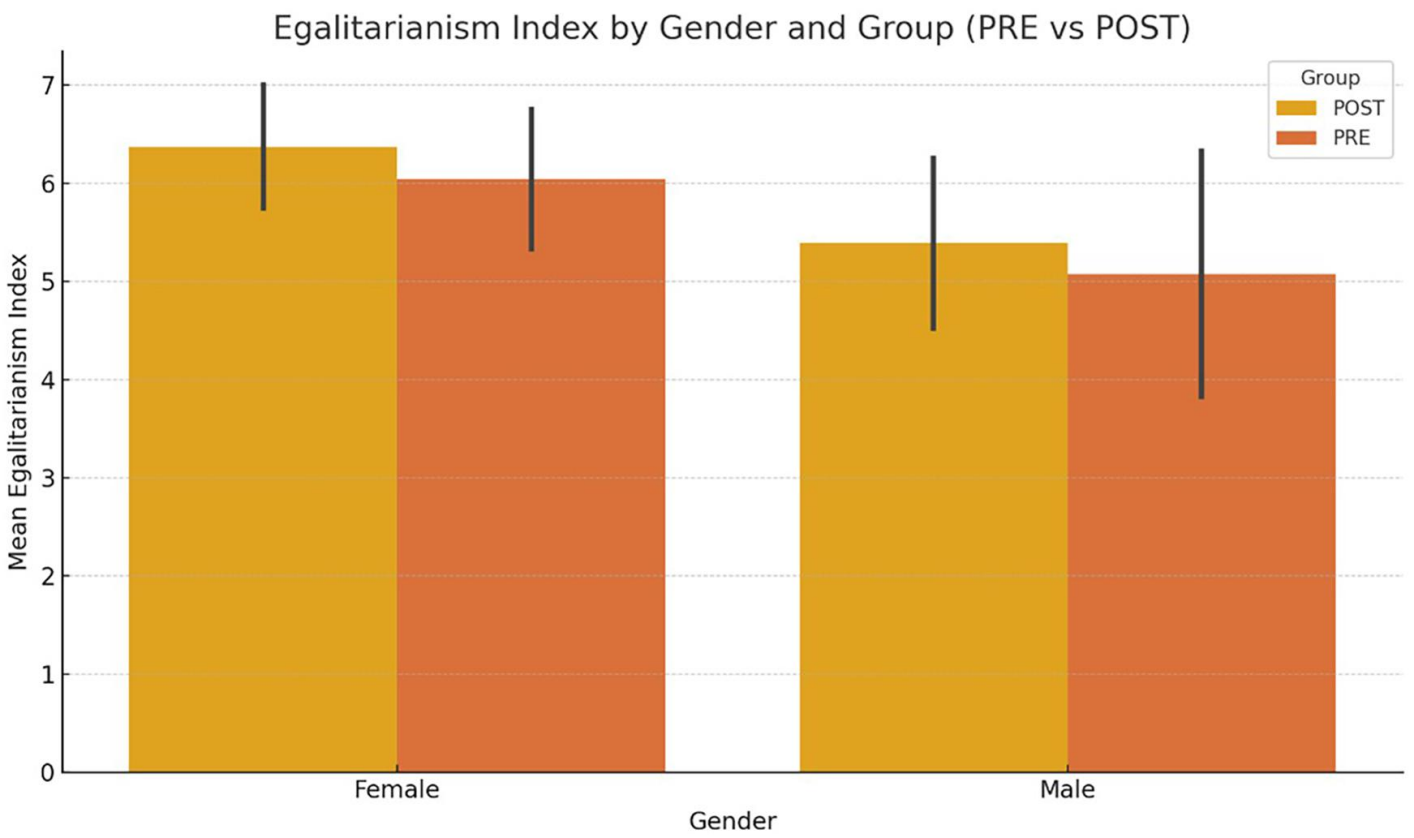
Visual representation of clusters and their characteristics.

The K-Means clustering analysis identified the following three clusters (Figure 1 shows it visually):

• Group 0: “Critical egalitarians”
  ○ Profile: This group shows a very favorable attitude towards gender equality (high rating of women's leadership skills, strong disagreement with negative stereotypes) and a high structural awareness of the gender problem at work.
  ○ Specific characteristics: They score very highly on: “Women have the ability to be good managers” (+1.03) and “The problem of inequality is structural” (+0.88). They score low on: “The possibility of becoming pregnant should influence management positions” (−2.18). They are very far to the left on the political spectrum (−3.12).
• Group 1: “Ambitious pragmatists”
  ○ Profile: This group shows strong professional aspirations (highest averages in willingness to work hard, promotion and leadership), but some skepticism or lukewarmness towards discourse on structural equality.
  ○ Specific characteristics: Very high motivation: “My professional goal is to progress” (6.5), “I have seriously considered taking on positions of responsibility” (6.5). Low score on “Female leadership ability” (−1.38). Lower perception of the structural gender problem (−0.70).
• Group 2: “Positive moderates”
  ○ Profile: This group maintains fairly egalitarian attitudes, although less radical than group 0. They also show a positive attitude towards their own health and physical fitness, and moderate professional ambition.
  ○ Specific characteristics: They score high on health and physical fitness (+0.75 and +0.89). Slightly in favour of gender equality (above average in several aspects). They are close to the political centre (+0.55).

This analysis was essential in designing the training workshop, its content and the most appropriate methodological strategies.

### 4.2 Workshops training impact (comparison between pre and post measurement)

An independent samples t-test was conducted to compare responses before (pre) and after (post) the intervention. The analysis included only items that were present in both datasets and measured on a numerical scale. There is no strong evidence that the impact of the intervention (pre vs. post) varies significantly depending on sociodemographic characteristics such as gender, health, or management experience. However, prior coordination experience may be associated with higher overall scores, suggesting a more favorable disposition toward the measured constructs.

Although none of the items show significant differences at the classical level of *p* < .05, two items are close to significance (*p* < .10), indicating a possible slight effect of the intervention on greater structural awareness of the gender problem and greater recognition of female capabilities:

• The lack of women in positions of power is a structural problem (*p* < .10).
• Women have the objectivity needed for business decisions (*p* < .10).

Although the difference is not statistically significant (*p* = 0.184), the post group shows a higher average Egalitarianism Index score (+0.51). This suggests a possible positive effect of the intervention on attitudes toward gender equality, especially considering the small sample size. In the post group, women show high and fairly consistent levels of egalitarianism (averages between 6.31 and 6.50).

**Figure 2:**
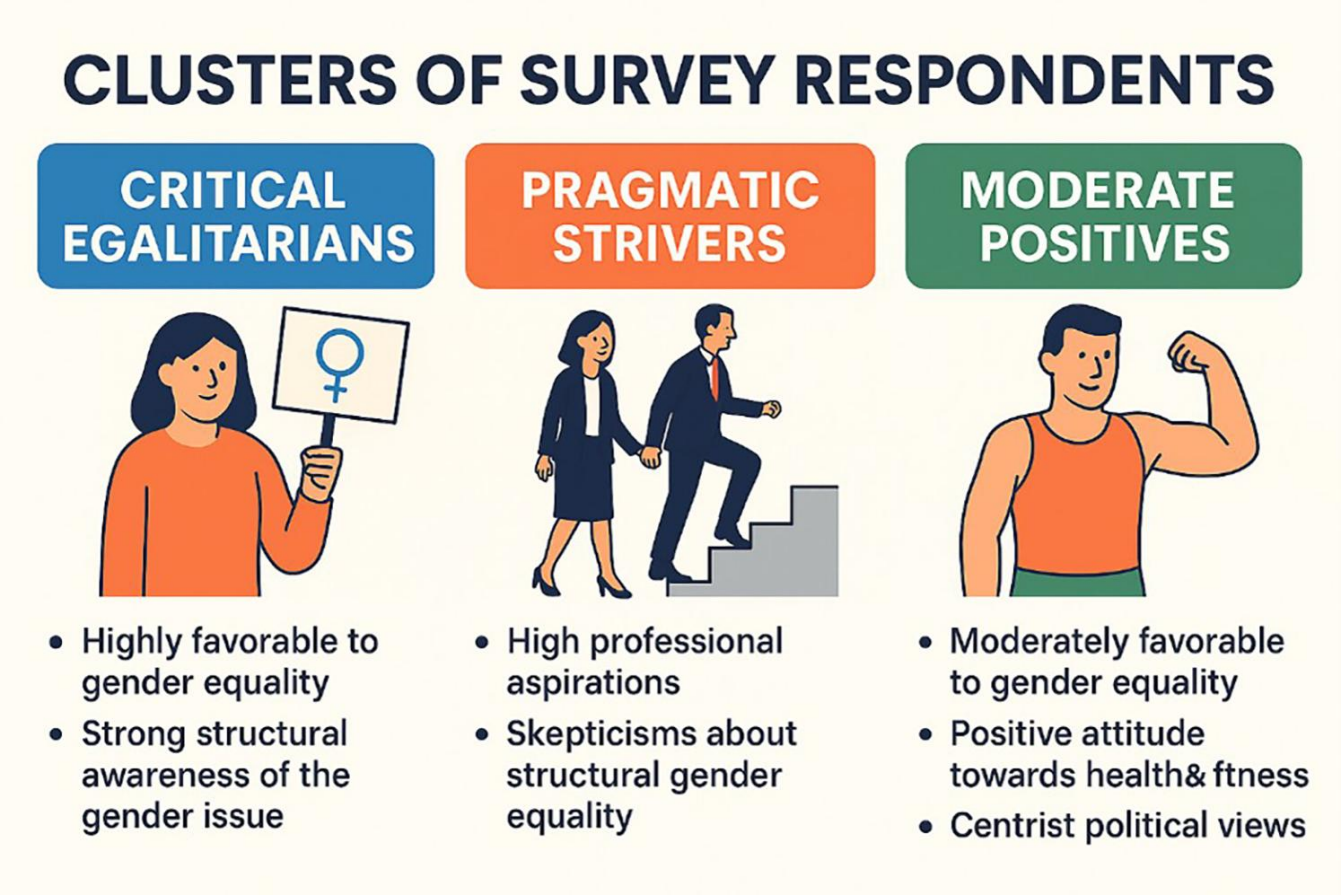
Egalitarianism index scores by gender comparison between pre and post measurements.

Figure 2 shows that, although not significant, there has been a positive change in the Gender Equality Index before and after the workshop for both men and women.

As can be seen, women in the post group have the highest average level of egalitarianism. In both groups, women score higher than men, although the difference is more pronounced in the post group. The change from pre to post is clearer in women than in men.

With regard to the clusters identified, those in the post-intervention group appear to be more concentrated, especially in the central area, suggesting a possible homogenisation of attitudes following the intervention. In contrast, in the pre-intervention group, the clusters are more dispersed, indicating greater diversity in initial profiles.

The new composition of the clusters after the intervention is as follows:

• Cluster 0 – “Conscious ambivalents”: Ideologically more to the left (average = 3). Strong disagreement with gender stereotypes. They recognise the structural problem (high egalitarianism), but show doubts about personal ambition (low score on “holding high positions”). High willingness to “work hard”, but mixed scores on leadership. Similar to pre Cluster 1, although now more critical of stereotypes.
• Cluster 1 – “Aspirational egalitarians”: Maximum support for structural equality, female objectivity, and personal ambition. Highly egalitarian and very motivated to take on leadership roles. Higher ideological level (10), but surprisingly progressive. Represents a powerful new profile compared to the pre one. Previously, there was biased leadership, but now equality and ambition are integrated.
• Cluster 2 – “Traditional with nuances”: More lukewarm support for equality (e.g., 5 on "structural problem"). They show residual stereotypes (3 in female inferiority, 4 in “pregnancy counts”). Low personal ambition. Very similar to Cluster 3 of the pre. Retains some resistance to change.

**Figure 3:**
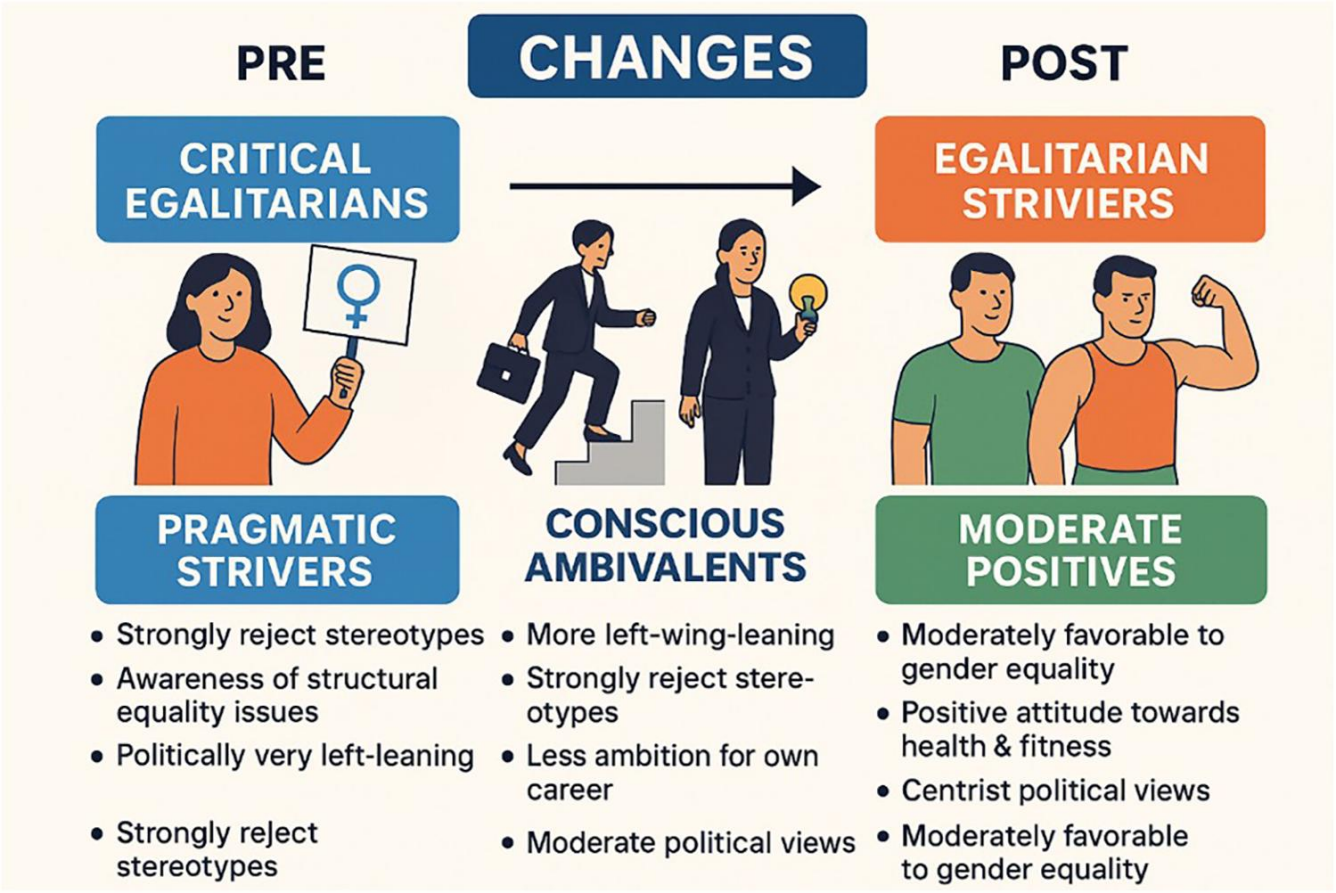
Differences between clusters before and after the training workshop.

Figure 3 displays the differences between clusters before and after the training workshop.

## 5 Discussion on the practical implications, objectives and lessons learned

Although the number of women in the sports workforce has increased, there is still significant room for improvement. This increase is necessary at all levels of the sports organisations, but in management areas it remains more visible. Moreover, it is well reported that gender equality has the power to transform society. In this regard, it is key to include content related to gender equality in the curriculum at all levels. The present project is a proposal for the implementation of gender equality content in the curriculum of the training programmes of sport studies.

Firstly, it needs to be highlighted that the results of the study show being male as one of the variables associated with lower egalitarian scores. Therefore, in a labour sector such as sports, where the majority of workers are male, it is imperative to develop training activities to achieve success in establishing gender equality content. In this sense, interventions such as the one in this paper are necessary, as they demonstrate a pilot experience on the impact that a training workshop on gender stereotypes – in this case, on leadership positions – can have on sports vocational training students.

One of the most significant findings is the gender difference in the evolution of attitudes. Women show greater awareness and support for equality, while men show more moderate attitudes. These results are consistent with other studies that highlight the importance of women's role as agents of change in initiatives that promote equality (17).

Although no significant differences were found in most of the items, the study results show a positive impact on students' attitudes toward gender equality in leadership positions, as reflected in the increase of the Egalitarianism Index (+0.51) after the intervention.

Based on the results obtained, it is evident that there is a need to raise awareness among the male gender about the importance of equality, which can be achieved through active and participatory methodologies. As previous studies show (16, 18, 32), structural barriers and gender stereotypes remain one of the main obstacles to women's access to leadership roles in sports. Therefore, in addition to including these contents at different educational levels, it is necessary to explore methodologies that foster awareness and promote attitudinal changes. The proposed approach uses participatory techniques such as brainstorming, gamified activities, and the use of “killer data,” which have proven to be useful pedagogical strategies for encouraging critical reflection and awareness, in line with Deterding et al. (21), and Hamari et al. (23).

On the other hand, traditionally, gender equality has been treated as a transversal content in the different subjects of the curriculum, and while it is a valid approach in primary and secondary school, it should be reinforced in higher levels through specific methodologies focused on building awareness of the matter, becoming an incentive for change.

The main limitations of this study are related to the aspects that make it a case study. On the one hand, the sample size does not allow for generalization of results and, on the other hand, the duration of the training. This study can therefore be considered a pilot for planning specific training courses on gender equality in leadership, which are so necessary, on an evidence-based basis, expanding the sample and the type of sports studies to which the instruments are applied.

For this reason, it is needed to add increase the number of similar experiences in order to evaluate the efficacy of the proposal, so repeating these activities in future promotions is key to achieving greater knowledge about how to improve the awareness and comprehension of the students about gender equality.

We must keep in mind the words of the IOC President, Thomas Bach, who states that “no organisation or country can afford to leave the skills of 50 per cent of the population behind – either in sport or in society at large” (31, 33).

## Data Availability

The raw data supporting the conclusions of this article will be made available by the authors, without undue reservation.
